# Variant Transcript of ROR1 ENST00000545203 Does Not Encode ROR1 Protein

**DOI:** 10.3390/biomedicines12071573

**Published:** 2024-07-16

**Authors:** Jie Xian, Navyaa Sinha, Christina Girgis, Christopher S. Oh, Matthew R. Cring, George F. Widhopf, Thomas J. Kipps

**Affiliations:** Center for Novel Therapeutics, Moores Cancer Center, Department of Medicine, University of California, San Diego, CA 92037, USA; j1xian@health.ucsd.edu (J.X.); nsinha@ucsd.edu (N.S.); csgirgis@ucsd.edu (C.G.); cso003@ucsd.edu (C.S.O.); mcring@ucsd.edu (M.R.C.); gwidhopf@ucsd.edu (G.F.W.II)

**Keywords:** ROR1, variant transcripts, neoplasia, anti-ROR1 antibody

## Abstract

Drs. John and Ford reported in *biomedicines* that a variant transcript encoding receptor tyrosine kinase-like orphan receptor 1 (ROR1), namely ENST00000545203 or variant 3 (*ROR1^V3^*), was a predominant *ROR1* transcript of neoplastic or normal cells in the Bioinformatic database, including GTEx and the 33 datasets from TCGA. Unlike the full-length *ROR1* transcript, Drs. John and Ford deduced that *ROR1^V3^* encoded a cytoplasmic ROR1 protein lacking an apparent signal peptide necessary for transport to the cell surface, which they presumed made it unlikely to function as a surface receptor for Wingless/Integrated (Wnt) factors. Moreover, they speculated that studies evaluating ROR1 via immunohistochemistry using any one of several anti-ROR1 mAbs actually may have detected cytoplasmic protein encoded by *ROR1^V3^* and that anti-cancer therapies targeting surface ROR1 thus would be ineffective against “cytoplasmic ROR1-positive” cancers that express predominately *ROR1^V3^*. We generated lentivirus vectors driving the expression of full-length *ROR1* or the *ROR1^v3^* upstream of an internal ribosome entry site (IRES) of the gene encoding a red fluorescent reporter protein. Although we find that cells that express *ROR1* have surface and cytoplasmic ROR1 protein, cells that express *ROR1^v3^* neither have surface nor cytoplasmic ROR1, which is consistent with our finding that *ROR1^v3^* lacks an in-frame initiation codon for ribosomal translation into protein. We conclude that the detection of ROR1 protein in various cancers cannot be ascribed to the expression of *ROR1^v3^*.

## 1. Introduction

Receptor tyrosine kinase-like orphan receptor 1 (ROR1) is a developmentally restricted type I glycoprotein expressed by a variety of different cancers. In *biomedicines*, Drs. John and Ford reported finding transcript variants of ROR1 upon examination of 34 transcriptomic datasets, including 33 cancer types and 54 non-diseased human tissues [1]. In addition to the full-length *ROR1* transcript, namely ENST00000371079, these investigators found a truncated variant transcript, called ENST00000545203 (designated here as *ROR1^v3^*), which has an 89 nucleotide-deletion in the 5′ region of full-length *ROR1*. Because this variant lacked the sequence encoding the signal peptide, Drs. John and Ford posited that *ROR1^v3^* encoded a cytoplasmic protein that was not expressed on the cell surface. As such, it would be unlikely that *ROR1^v3^* encoded a ROR1 protein that could serve as a ligand for exogenous windless integration site (Wnt) factors or that could be targeted by anti-ROR1 monoclonal antibodies (mAbs) or chimeric antigen receptor (CAR) T cells. Also, because *ROR1^v3^* appeared to represent the predominant transcript in some tumor samples, they speculated that prior studies evaluating ROR1 via immunohistochemistry using any one of several anti-ROR1 mAbs actually may have detected cytoplasmic rather than surface ROR1 [2,3,4,5,6]. However, they did not determine whether *ROR1^v3^* actually could encode a cytoplasmic protein, prompting us to examine for cytoplasmic ROR1 in cells made to express *ROR1^v3^* (ENST00000545203).

## 2. Materials and Methods

### 2.1. Cell Line

MEC1 cells were purchased from ATCC and maintained in RPMI with 10% FBS, 2 mM L-glutamine, 20 units/mL penicillin and 20 mg/mL streptomycin. Suspension HEK293-derived Viral Production Cells were obtained from Thermo Fisher Scientific (Waltham, MA, USA) and cultured in LV-MAX Lentiviral Production medium (Catalog A35684, Gibco, Emeryville, CA, USA). Various derivative cell lines were made in the present study. All cells were cultured in a humidified atmosphere with 5% CO_2_ at 37 °C. All cell lines tested negative for mycoplasma by MycoAlert detection kit (Catalog LT07-118, Lonza Biologics, Bend, OR, USA).

### 2.2. Plasmid Construction

pLV-EF1a-ROR1 was constructed by inserting ROR1 expression gene into a pLV-EF1a backbone (Catalog 85132, Addgene, Watertown, MA, USA) using NEBuilder HiFi DNA Assembly (Catalog E5520, NEB, Ipswich, MA, USA) using a donor ROR1-expressing plasmid, pcDNA3.1-ROR1, which is a plasmid we previously generated by inserting the *ROR1* ORF (Catalog RG214967, Origene Technologies, Rockville, MD, USA) into pcDNA3.1 (Catalog V79020, Invitrogen, Carlsbad, CA, USA). For RFP expression, an IRES-dTomato gBlock was inserted into the pLV-EF1a-ROR1 using NEBuilder HiFi DNA Assembly (Catalog E5520, NEB) to produce pLV-EF1a-ROR1-IRES-dTomato. *ROR1^v3^* transcript plasmid was constructed by modifying pLV-EF1a-ROR1-IRES-dTomato using Q5 Site-Directed Mutagenesis (Catalog E0554, NEB) according to the manufacturer’s protocol.

### 2.3. Lentivirus Production and Transduction

LV-MAX Lentiviral Production System (Catalog A35684, Gibco) was used to package lentivirus. A total of 37.5 μg transfer plasmid together with packaging plasmids (21.2 μg of pMD2.G, 9.96 μg of pRSV-Rev and 13.85 μg of pMDLg/pRRE) were transfected into 30 mL of suspension HEK293-derived Viral Production Cells at a concentration of 4 million/mL per the manufacturer’s protocol. The kit enhancer was added after 6 h, and viral supernatant was collected 48 h post-transfection, centrifuged at 1500× *g* for 10 min, passed through a 0.45 μm filter to remove the cell debris, aliquoted and stored at −80 °C. Lentivirus was used to transduce MEC1 cells with synperonic F108 (100 μg/mL), followed by FACS sorting gated on the positive red fluorescent signal of dTomato.

### 2.4. Reverse-Transcription PCR

Total RNA was extracted using TRIzol (Catalog 15596018, Life Technologies, Carlsbad, CA, USA). A total of 10 μg of total RNA was incubated with 10 U RNase-free DNase I (Catalog EN0521, Life Technologies) at 37 °C for 30 min. RNA was further purified using the RNeasy Mini Kit (Catalog 74104, QIAGEN, Germantown, MD, USA). The purified total RNA (2 μg) was used for qPCR per the Luna^®^ Universal One-Step RT-qPCR Kit (Catalog E3005S, NEB) protocol. The primers used were as follows:

ROR1 F 5′-ACCGCACCGTGTATATGGAGTCT-3′;

R 5′-GCATAGTGGCACAGGGAAGG-3′;

GAPDH F 5′-GTCTCCTCTGACTTCAACAGCG-3′;

R 5′-ACCACCCTGTTGCTGTAGCCAA-3′.

### 2.5. Immunoblot

Western blot analysis was performed as described in [3,4]. Briefly, cells were lysed by RIPA buffer containing cOmplete Protease inhibitor (Catalog 11697498001, Roche, Pleasanton, CA, USA) and PhosSTOP phosphatase inhibitor (Catalog 4906845001, Roche), followed by a centrifugation step of 1500× *g* for 20 min at 4 °C. Equal amounts of total protein from each sample were fractionated by SDS-PAGE and blotted onto polyvinylidene difluoride membrane. Western blot analysis was performed using primary mAbs specific for ROR1 (1:1000 dil, catalog 4102, Cell Signaling Technology, Danvers, MA, USA), ROR1 (1:500 dil, catalog ROR1-101AP, FabGennix, Frisco, TX, USA), ERK1/2 (1:1000 dil, catalog 9102, Cell Signaling Technology), phosphoERK1/2 (1:1000 dil, catalog 9101, Cell Signaling Technology), or β-actin (1:1000 catalog 4967, Cell Signaling Technology), which were detected using anti-rabbit IgG HRP-linked secondary antibody (1:2000 dil, catalog 7074, Cell Signaling Technology). Blots were developed using SuperSignal™ West Femto Maximum Sensitivity Substrate (Catalog 34094, Thermo Fisher), and imaged using ChemiDoc Imaging Systems (BioRad, Irvine, CA, USA). Band densities were measured using the Image J 1.42q software (NIH, Bethesda, MD, USA).

### 2.6. Statistical Analysis

Statistical analysis was carried out using GraphPad Prism software v8 (GraphPad Software, La Jolla, CA, USA) and *p*-values were determined using the unpaired two-way ANOVA test and considered significant with a *p*-value of less than 0.05.

## 3. Result

**Figure 1:**
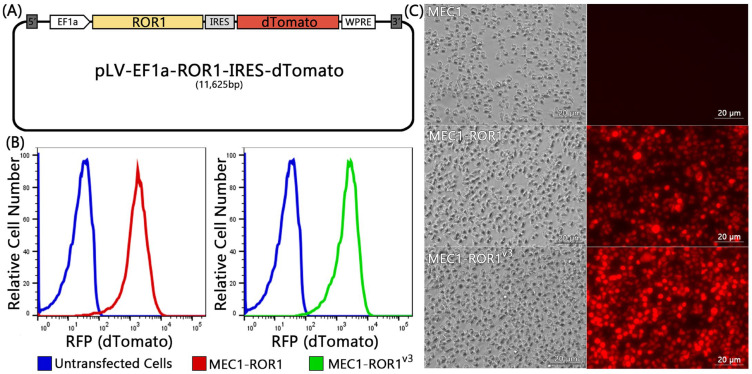
(**A**) ROR1 lentiviral plasmid map containing full-length *ROR1* or *ROR^v3^* upstream of an IRES and gene encoding RFP, namely dTomato. MEC1, MEC1-ROR1, or MEC1-ROR1^v3^ were examined for RFP fluorescence by (**B**) flow cytometry cells or (**C**) fluorescence microscopy.

We generated lentivirus vectors encoding full-length *ROR1* or *ROR1^v3^* upstream of an internal ribosome entry site (IRES) of the gene encoding the red fluorescent protein, namely dTomato, a protein with red fluorescence when excited by green–yellow light. This allowed us to monitor the transduction efficiency of each lentivirus vector for MEC1 cells, a human leukemia cell line lacking endogenous ROR1 (Figure 1A) [7]. We found that MEC1 cells transduced with either ROR1-RFP or ROR1^v3^-RFP each acquired red fluorescence, as assessed via flow cytometry (Figure 1B) or fluorescence microscopy (Figure 1C).

**Figure 2:**
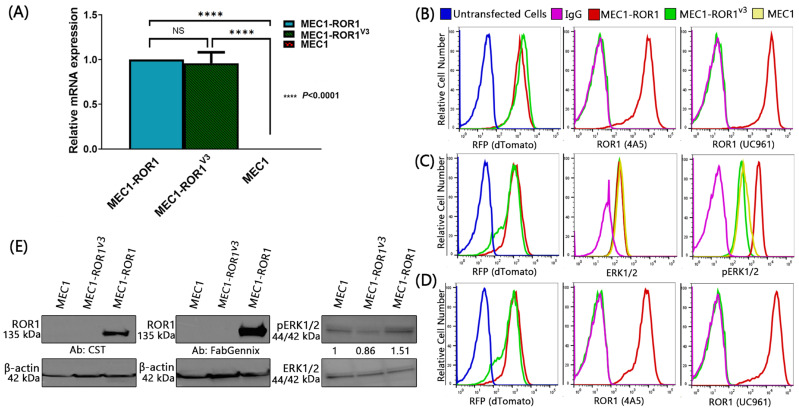
*ROR1* mRNA and protein detection. (**A**) *ROR1* mRNA expression analysis by RT-PCR. Two-way ANOVA was used to determine statistical significance for mRNA expression. Bar graphs represent data summarized as mean ± S.E.M. and were analyzed by unpaired two-sided Mann–Whitney test. Data shown are representative of three experiments. NS (non-significant). (**B**) ROR1 cell surface staining was assessed via flow cytometry using fluorochrome labeled anti-ROR1 mAb 4A5 or UC-961. (**C**) Fixed and permeabilized cells were stained with an Alexa-488-conjugated mAb specific for ERK1/2 and an Alexa-647-conjugated mAb specific for phosphorylated ERK1/2 and then examined via flow cytometry. (**D**) Fixed and permeabilized cells were stained with Alexa-647-conjugated anti-ROR1 mAbs, 4A5 or UC961, and then analyzed via flow cytometry, as indicated in the figure. (**E**) Immunoblot of cell lysates of MEC1, MEC1-ROR1, or MEC1-ROR^v3^ probed for ROR1 using CST antibody (CST ROR1 Cat No: #4102, left) or FabGennix antibody (FabGennix Cat No: ROR1-101AP, right). The uncropped blots are shown in Supplementary Materials.

We examined the cDNA of these transduced MEC1 cells by qPCR for expression of full-length *ROR1* or *ROR1^v3^* transcripts. We found that MEC1 cells transduced with either vector had comparable levels of full-length *ROR1* or *ROR1^v3^* transcripts (Figure 2A). As expected, MEC1 cells transduced with the lentivirus encoding ROR1, but not MEC1 cells transduced to express ROR1^v3^, expressed surface ROR1, as assessed via flow cytometry using either of two fluorochrome-conjugated mAbs specific for either of two epitopes of extracellular ROR1 (Figure 2B). In addition, despite having comparable levels of ERK1/2, we detected pERK1/2 in MEC1 cells that expressed *ROR1,* but not in MEC1 expressing *ROR1^v3^,* by phospho-flow cytometry (Figure 2C). However, we also did not detect cytoplasmic ROR1 in MEC1 cells transduced to express *ROR1^v3^* with either of the two mAbs (Figure 2D).

We assessed for ROR1 protein in cell lysates of MEC1, MEC1-ROR1, or MEC1-ROR^v3^ cells via immunoblot analyses using either of two additional anti-ROR1 antibodies specific for distinct epitopes of ROR1, namely anti-ROR1 #4102 Cell Signaling Technology, which binds a putative extracellular epitope of ROR1, and FabGennix #ROR1-101AP, which binds an intracellular epitope of ROR1 (aa positions 900–918). However, neither of these mAbs detected ROR1 protein in lysates of MEC1-ROR1^v3^ (Figure 2E).

## 4. Discussion

ROR1 is a developmentally restricted type I surface glycoprotein that is expressed on some embryonic cells and neoplastic cells of a variety of different cancers, but virtually not on normal adult tissue. ROR1 can serve as a surface receptor for Wnt5a, which can induce ROR1 signaling, leading to activation of ERK1/2, NF-κB, and NRF2, and enhance cancer stemness [8]. Moreover, the expression of ROR1 by cancer cells has been associated with enhanced cancer cell migration, epithelial–mesenchymal transition (EMT), increased risks of relapse and/or metastasis, and an unfavorable prognosis relative to that of cancers that do not express ROR1 [9,10,11,12]. This is posited as being secondary in part to Wnt5a-induced ROR1 signaling [8]. Consistent with this notion are data showing that treatment of patients with chronic lymphocytic leukemia (CLL) with anti-ROR1 mAb that can block ROR1 signaling, e.g., zilovertamab, formerly called cirmtuzumab or UC-961, significantly reduced cancer stemness and leukemia-cell expression of target genes induced by activated ERK1/2, NF-κB, and NRF2 relative to that of leukemia cells prior to therapy [13,14,15,16]. The distinctive expression of ROR1 and its putative role in cancer progression has stimulated development and testing of agents that can target surface ROR1 for the treatment of patients with cancer, such as anti-ROR1 mAbs, antibody–drug conjugates (ADC), bispecific antibodies, and CAR T cells, as well as small-molecule inhibitors (reviewed in [8]).

However, in a recent paper published in *biomedicines* by Drs. John and Ford, the authors challenged the notion that targeting surface ROR1 may be an effective therapy for all patients with cancers found to express *ROR1* or to stain positive for ROR1 with anti-ROR1 mAbs via immunohistochemistry (IHC) [1]. They interrogated a Bioinformatic database, which included the Genotype-Tissue Expression (GTEx) Portal and the 33 datasets from the Cancer Genome Atlas (TCGA), and identified variant truncated isoforms of ROR1 that were also expressed in some normal and neoplastic cells. In particular, they noted that one variant ROR1 transcript, ENST00000545203, which they called variant 3 (*ROR1^V^*^3^), was expressed as equal or greater levels than the full-length *ROR1* transcript. Moreover, as *ROR1^V^*^3^ featured an 89-nucleotide deletion in the 5′ region of *ROR1*, they speculated that it encoded a ROR1 protein lacking a leader sequence, which they assumed would preclude it from being expressed as a surface membrane protein, but rather relegate it to being a cytoplasmic protein. Accordingly, they reasoned that cancer cells expressing predominately *ROR1^V3^* relative to *ROR1* would predominately express cytoplasmic, and not surface, ROR1 that could react with anti-ROR1 mAbs by IHC. If so, then such cancer cells found to express ROR1 by IHC, as noted by others [2,17,18,19,20,21,22,23,24,25,26], may not be targeted by agents intended to react with cells bearing surface ROR1 protein [1].

In the current study, we examined whether *ROR1^V3^* could encode a truncated cytoplasmic ROR1 protein. To monitor for the effectiveness of transduction, we generated bicistronic vectors with a downstream fluorescence reporter gene that encoded dTomato, which could be detected via fluorescence microscopy or flow cytometry (Figure 1). We confirmed that *ROR1* encoded a protein that could be detected on the surface of a leukemia-cell line, MEC1. Despite expressing similar amounts of dTomato as cells transduced with the bicistronic vector housing *ROR1*, the MEC1 cells transduced with the bicistronic vector housing *ROR1^V3^* failed to make detectable surface or cytoplasmic ROR1 protein (Figure 2B–E). As MEC1 cells constitutively make ample quantities of Wnt5a, we were able to examine for ROR1 signaling in MEC1 cells transduced to express either *ROR1* or *ROR1^V3^*. As expected, although MEC1-ROR1 cells had an increased activation of ERK1/2 than MEC1 cells, we did not see an increased activation of ERK1/2 in MEC1 cells made to express *ROR1^V3^* (Figure 2E), which we could readily detect in MEC1-ROR1^V3^ via RT-PCR (Figure 2A). These studies refute the notion advanced by Drs. John and Ford that many tumors found to react with anti-ROR1 mAb by IHC predominantly may express a cytoplasmic protein encoded by *ROR1^V3^*.

It is possible that *ROR1^V3^* plays a functional role, serving as a so-called *sterile transcript* in post-transcriptional mechanisms regulating gene expression into protein. Most notable are microRNAs, which can bind to complementary sequences in the 3′ untranslated region (UTRs) of target mRNAs, thereby leading to translational repression or mRNA degradation [27,28]. In particular, *miR-15a/16-1* can target *ROR1*, thereby repressing its expression [29]. Conversely, the deletion of *miR-15a/16-1*, as noted in CLL, can affect upregulation in the expression of *ROR1* and its encoded protein [29]. In a similar fashion, truncated sterile transcripts of *ROR1*, lacking the 5’ region, but still possessing the complementary sequences targeted by such microRNA, may instead act as a molecular ‘sponge’, thereby repressing the capacity of microRNA such as *miR-15a/16-1* to repress expression of the full-length *ROR1* transcript. If so, then we speculate that a high-level expression of *ROR1^V3^* actually may enhance the expression of ROR1 protein by cells that express relatively low levels of the full-length *ROR1* by competing for microRNA that ordinarily might repress the expression of *ROR1* or its translation into surface ROR1 protein.

In any case, our findings reported here are consistent with the noted lack of an in-frame translational start codon in *ROR1^v3^* allowing for a truncated ROR1 protein to be produced from a cryptic start site. We conclude that the ROR1 transcript variant ENST00000545203 does not encode a ROR1 protein, as evidenced by multiple protein detection methodologies. Given these findings, further functional research should focus instead on the full-length ROR1 protein encoded by the full-length *ROR1* transcript.

## Data Availability

The datasets used and/or analyzed during the current study are available from the corresponding author on reasonable request.

## Supplementary Materials

The following supporting information can be downloaded at: https://www.mdpi.com/article/10.3390/biomedicines12071573/s1.
