# Supplementary material for: Variant Transcript of ROR1 ENST00000545203 Does Not Encode ROR1 Protein
Source: Biomedicines. 2024 Jul 16;12(7):1573. doi: 10.3390/biomedicines12071573 (PMC11274362; doi:10.3390/biomedicines12071573)
Supplement: Supplementary file 1 [file biomedicines-12-01573-s001.zip › biomedicines-3078406-supplementary.pptx]

## Slide 1
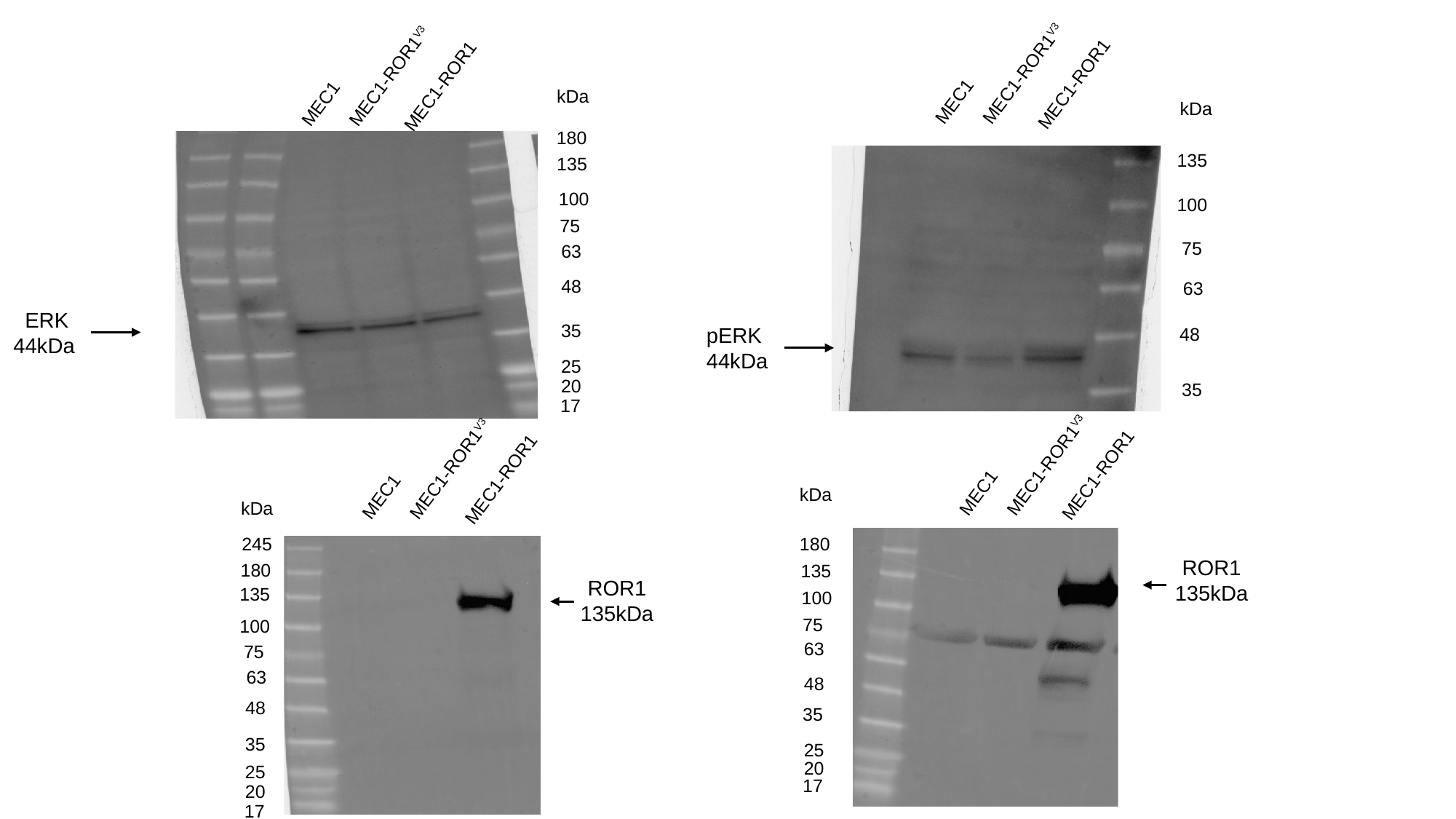

MEC1-ROR1V3
MEC1-ROR1V3
MEC1-ROR1
MEC1-ROR1
MEC1
MEC1
kDa
kDa
180
135
135
100
100
75
75
63
48
63
ERK
44kDa
35
pERK
44kDa
48
25
20
35
17
MEC1-ROR1V3
MEC1-ROR1V3
MEC1-ROR1
MEC1-ROR1
MEC1
MEC1
kDa
kDa
180
245
ROR1
135kDa
180
135
ROR1
135kDa
135
100
75
100
63
75
63
48
48
35
35
25
20
25
17
20
17

## Slide 2
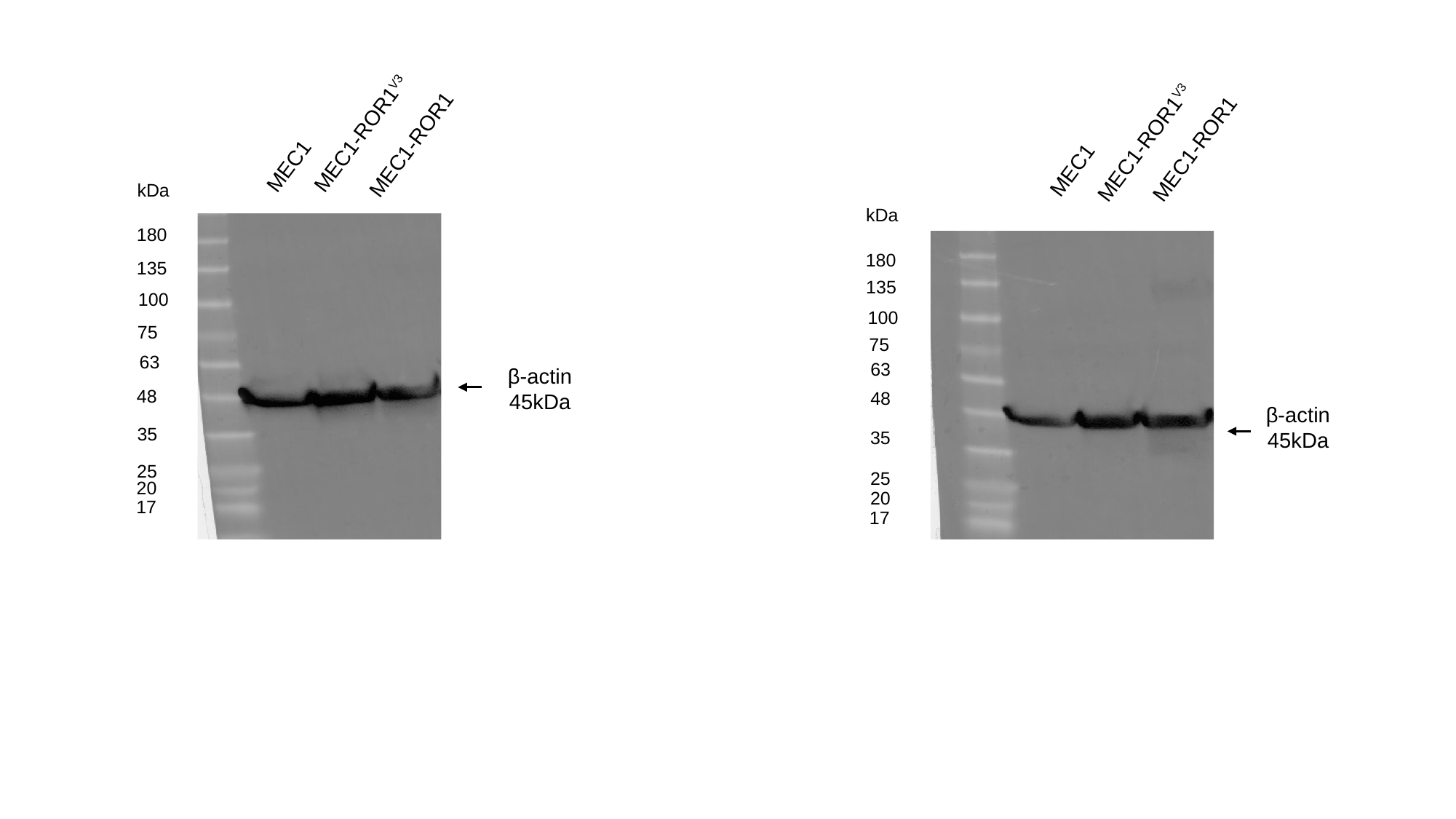

MEC1-ROR1V3
MEC1-ROR1V3
MEC1-ROR1
MEC1-ROR1
MEC1
MEC1
kDa
kDa
180
180
135
135
100
100
75
75
63
63
β-actin
45kDa
48
48
β-actin
45kDa
35
35
25
25
20
20
17
17

## Slide 3
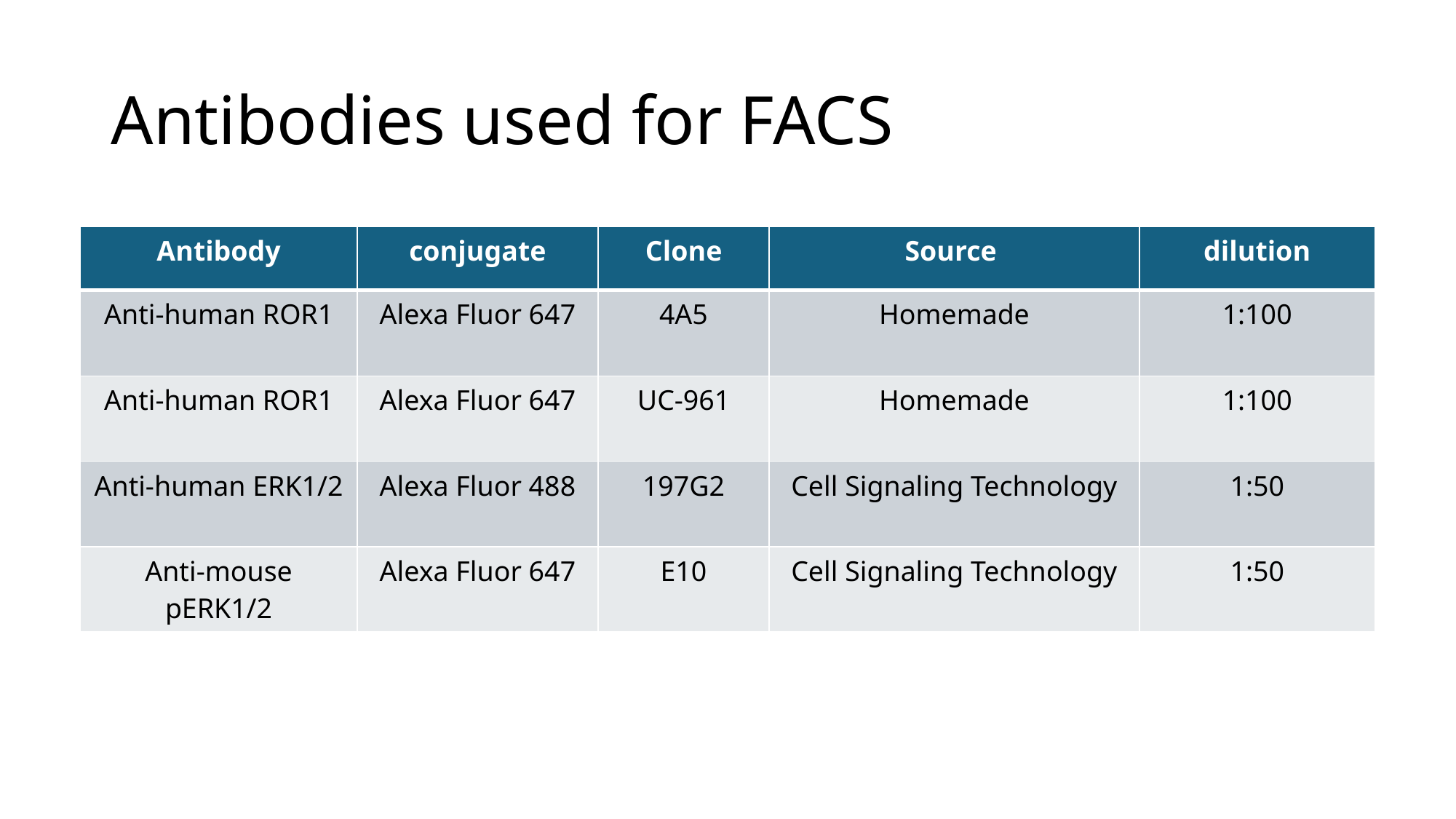

# Antibodies used for FACS
| Antibody | conjugate | Clone | Source | dilution |
| --- | --- | --- | --- | --- |
| Anti-human ROR1 | Alexa Fluor 647 | 4A5 | Homemade | 1:100 |
| Anti-human ROR1 | Alexa Fluor 647 | UC-961 | Homemade | 1:100 |
| Anti-human ERK1/2 | Alexa Fluor 488 | 197G2 | Cell Signaling Technology | 1:50 |
| Anti-mouse pERK1/2 | Alexa Fluor 647 | E10 | Cell Signaling Technology | 1:50 |
